# Best Practices for Including Individuals With Dementia as Research Participants

**DOI:** 10.1093/geroni/igaa057.906

**Published:** 2020-12-16

**Authors:** Katherine Judge, Morgan Minyo, Amanda MacNeil, Claire Grant, Kaitlyn Lucas

**Affiliations:** Cleveland State University, Cleveland, Ohio, United States

## Abstract

Including individuals with dementia as participants in research studies can be a challenge given the cognitive symptoms experienced, including difficulties with attention, memory, language, and executive functioning and behavioral symptoms, such as agitation and frustration. Researchers, however, have begun to include individuals with dementia as more active participants in the research process. Specifically, individuals with dementia have provided self-report information about their illness experience, including how they cope and manage with their illness and how illness-related strains are related to psychosocial outcomes. Researchers also have starting using self-report data collection protocols instead of proxy-report measures to assess the impact of non-pharmacological interventions developed for individuals with dementia on well-being outcomes such as depressive symptoms, unmet needs, and relationship strain. To date, however, there are no clear guidelines or ‘best practices’ to assist researchers in developing study protocols that facilitate the inclusion and participation of individuals with dementia. This poster will present findings from a systematic literature review of studies that have successfully included individuals with dementia as research participants. Key areas examined include: 1) the focus and type of research question studied; 2) the use and type of cognitive screening tools for determining study eligibility; 3) implementation of qualitative versus quantitative research designs; 4) objective versus subjective measures; 5) data collection tools; 6) psychometric evidence of data obtained; and 7) how data have been analyzed. Findings highlight the importance of collecting data directly from individuals with dementia to further understand the illness experience and assess the impact of non-pharmacological interventions.

